# Case Report: Low Hematocrit Leading to Tacrolimus Toxicity

**DOI:** 10.3389/fphar.2021.717148

**Published:** 2021-08-16

**Authors:** Alexandre Piletta-Zanin, Aurélie De Mul, Nathalie Rock, Pierre Lescuyer, Caroline F. Samer, Frédérique Rodieux

**Affiliations:** ^1^ Division of Clinical Pharmacology and Toxicology, Department of Anesthesiology, Pharmacology, Intensive Care and Emergency Medicine, Geneva University Hospitals, Geneva, Switzerland; ^2^ Division of Pediatric Specialties, Department of Women, Children and Adolescents, Geneva University Hospitals, Geneva, Switzerland; ^3^ Pediatric Nephrology Unit, Department of Women, Children and Adolescents, Geneva University Hospitals, Geneva, Switzerland; ^4^ Swiss Pediatric Liver Center, Department of Women, Children and Adolescents, Geneva University Hospitals, Geneva, Switzerland; ^5^ Division of Laboratory Medicine, Department of Diagnostic, Geneva University Hospitals, Geneva, Switzerland; ^6^ Faculty of Medicine, University of Geneva, Geneva, Switzerland

**Keywords:** tacrolimus, monitoring, hematocrit, toxicity, subtherapeutic, pharmacokinetic, ABCB1, pharmacogenetic

## Abstract

Tacrolimus is a calcineurin inhibitor characterized by a narrow therapeutic index and high intra- and inter-individual pharmacokinetic variability. Therapeutic drug monitoring in whole-blood is the standard monitoring procedure. However, tacrolimus extensively binds to erythrocytes, and tacrolimus whole-blood distribution and whole-blood trough concentrations are strongly affected by hematocrit. High whole-blood tacrolimus concentrations at low hematocrit may result in high unbound plasma concentrations and increased toxicity. We present the case of a 16-year-old girl with kidney and liver transplant in whom low concentrations of tacrolimus in the context of low hematocrit led to significant increase in the dosage of tacrolimus and participate, along with a genetic polymorphism of *ABCB1*, in nephrotoxicity.

## Introduction

Tacrolimus is one of the most frequently prescribed immunosuppressant after solid organ transplant. Because of its narrow therapeutic index, tacrolimus concentrations have to be maintained within the therapeutic margin to achieve therapeutic immunosuppression and minimize toxicity (Böttiger et al., 1999; Astellas Pharma US, 2012). Therapeutic drug monitoring (TDM) of tacrolimus trough concentrations (C_t_) performed in whole-blood is the standard procedure (Böttiger et al., 1999; Astellas Pharma US, 2012).

High inter-individual variability in tacrolimus pharmacokinetic (PK) parameters and C_t_ is observed after administration of a fixed dose of tacrolimus. Several clinical factors may influence the PK of tacrolimus, such as type of organ transplanted, time since transplantation, age, sex, food intake, concomitant treatments, liver and kidney dysfunction, as well as genetic factors such as polymorphisms of the cytochromes P450 (CYP) 3A4/5 and drug transporter P-glycoprotein (P-gp). As tacrolimus extensively binds to erythrocytes, tacrolimus whole-blood distribution and tacrolimus whole blood concentration (TWBC) are strongly affected by hematocrit (Hct) (Staatz et al., 2010).

We report, for the first time, the case of a pediatric patient who developed severe tacrolimus toxicity despite subtherapeutic TWBC, due to low Hct levels.

## Case

A 16-year-old girl known to have autosomal recessive polycystic kidney disease with end-stage renal disease and liver fibrosis received liver and kidney transplant, from the same deceased donor. The postoperative immunosuppressive regimen consisted of intravenous (IV) basiliximab (Simulect®), corticosteroids (methylprednisone, prednisone), IV mycophenolate mofetil (Cellcept®) and oral immediate-release tacrolimus (Modigraf®). From the day following transplantation (day 1), tacrolimus was given through a nasogastric tube as granules for oral suspension at the initial dosing regimen of 5 mg/d (0.1 mg/kg/d) in two daily doses, followed by dose adjustments to reach the target TWBC of 7–12 μg/l (Roche Elecsys® electro-chemiluminescence immunoassay–ECLIA), as recommended in our institution. Mycophenolate mofetil was given at a dose of 2 g/d. Steroids were given as follows: IV methylprednisone 500 mg during surgery, 250 mg on the first postoperative day and 125 mg on day 2, followed by oral prednisone with a reduction regimen starting on day 3 at a dose of 80 mg/d as standard protocol for liver and renal transplantation in our institution. Other standard transplantation treatment consisted of IV piperaciline-tazobactam followed by oral trimethoprim/sulfamethoxazole, IV ganciclovir, oral omeprazole, oral labetalol, and IV acetaminophen. A nasogastric tube was used to administer oral drugs.

The post-operative period was complicated by significant anemia due to hemorrhage during hepatectomy, requiring blood transfusions at day 3 and intracerebral hemorrhage with intracranial hypertension requiring sedation with IV propofol, IV midazolam, IV morphine, IV rocuronium from day 8 to day 19, as well as oral levetiracetam. An infected biloma was diagnosed on day 15 by computed tomography with intravenous contrast and treated by IV meropenem.

TWBC were highly variable during the first 10 postoperative days; concentrations ranged from 6.1 to 24 µg/l with a dose of 2.4–5 mg/d. From day 11 to day 16, TWBC remained subtherapeutic despite the increase in dose up to 12 mg/d (see Table 1). On day 17 and 18, a therapeutic TWBC of 8.4 µg/l was finally achieved with a dose of 10 mg/d. On day 18, the patient developed a Stage 1 acute kidney injury based on Kidney Disease Improving Global Outcome (KDIGO) consensus [Kidney Disease: Improving Global Outcomes (KDIGO) Transplant Work Group, 2012; Levey et al., 2020]. From day 19, due to a suspicion of graft rejection, the dose was increased to 14 mg/d; on day 20 methylprednisone 600 mg/d was introduced and TWBC remained subtherapeutic. Renal biopsy on day 20 showed no sign of graft rejection (including negative CD4 staining), but confirmed severe acute tubular necrosis. On day 23, the patient had other signs consistent with tacrolimus toxicity such as hyperglycemia requiring insulin treatment, resistant hypertension treated with oral labetolol and oral minoxidil, and tremors. On day 23, despite the persistence of subtherapeutic TWBC, in order to limit nephrotoxicity, tacrolimus dosage was decreased to 10 mg/d.

**TABLE 1:** Patient’s laboratory values and relevant clinical evolution.

Post-operative day	Clinical evolution		Tacrolimus dose	Plasma creatinine	GFR (modified Schwartz)	Hct	TWBC (ECLIA)	Predicted TWBC for 40% Hct
		Units	mg/d	44–80 µmol/l	ml/min/1.73m2	%	µg/l	µg/l
		Target/ref ranges				36–46	7–12	
d 0	*Liver and kidney transplant*							
d 11			4.4	114	52	24.6	6.1	10.5
d 12			5.4	91	65	25.2	6.4	10.8
d 13			6	84	71	24.1	7.9	12.5
d 14			8	77	77	24.2	5.4	8.5
d 15			12	67	89	25.4	2.8	5
d 16			10	61	98	24.4	7.1	11.2
d 17			9	64	93	24	8.4	**15**
d 18	*Acute kidney injury (stage 1)*		10	92	65	23.4	8.4	**15**
d 19	*Suspicion of graft rejection*		12	108	55	24.2	5.8	9.8
d 20	*No signs of graft rejection (renal biopsy)*		14	110	54	23.5	3.9	7.3
d 22			14	114	52	21.5	5.7	11.5
d 23	*Tremor, hypertension, hyperglycemia*		10	116	52	19.6	5.8	11.5
d 24			10	115	52	24.6	5.4	8.7
d 25			10	103	58	24.9	4.6	7.3
d 26			9	97	61	24.6	6.4	11.5
d 27			8	88	68	25.3	4.9	8
d 30	*Kidney recovery*							

Bold values indicates the highly supratherapeutic concentrations.

Investigations were conducted to assess the causes of tacrolimus toxicity despite a subtherapeutic TWBC. Patient’s medical compliance was adequate and she was not taking any over-the-counter medications. No PK interactions or the presence of the biloma could explain the decreased TWBC concentrations and signs of toxicity. TWBC measured by ECLIA were confirmed using a liquid chromatography-mass spectrometry (LC/MS) method to exclude an analytical interference. The potential inducing effect of methylprednisolone and prednisone on CYP3A (Anglicheau et al., 2003) was not correlated with the sudden drop in TWBC. *CYP3A4, CYP3A5* and *ABCB1* genotyping (c. 3435T>C and c.2677T>G/A) were performed on the DNA of the donor and recipient. CYP3A phenotyping was performed on the recipient with midazolam as a probe drug and measurement of the OH-midazolam/midazolam metabolic ratio. The donor and the recipient had CYP3A4 normal activity and were CYP3A5 non expressors. Genotyping of the *ABCB1* gene encoding P-glycoprotein (P-gp) revealed that the donor was a heterozygous carrier of the c. 3435T>C polymorphism. Although this genetic polymorphism is associated with a decrease in P-gp activity of the transplanted organ, no study has shown an impact of this genotype on tacrolimus concentrations (Yan et al., 2016).

**FIGURE 1:**
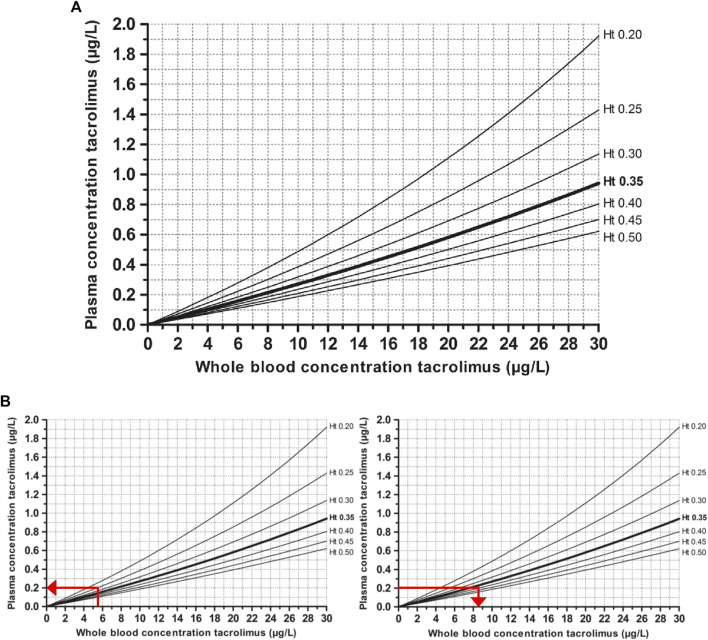
Nomograph of predicted tacrolimus plasma concentrations corrected for hematocrit. **(A)** Schijvens’ nomograph **(B)** Use of the Schijvens’ nomograph to convert the measured TWBC (5.4 µg/l with 24.6% Hct) on day 24 to **[B(i)]** predicted tacrolimus plasma concentration and **[B(ii)]** predicted TWBC for 40% Hc.

Because of a persistent anemia with an Hct value of less than 25%, the nomograph of “predicted tacrolimus plasma concentrations corrected for Hct” published by Schijvens et al. (2019), which described the relationship between TWBC and plasma tacrolimus concentrations for different Hct levels, was used to correct the TWBC (see Figure 1A). Predicted TWBC for 40% Hct revealed supratherapeutic concentrations (15 µg/l) on day 17 and 18 as well as high concentrations (11.5 µg/l) on day 22 and 23. Tacrolimus plasma concentrations were measured on day 24 by LC/MS and compared to the tacrolimus plasma concentrations predicted by the nomograph to assess the method’s accuracy (Figure 1B).

Based on the biopsy, clinical and laboratory findings, and retrospective determination of the predicted TWBC based on Hct values, we concluded that despite low tacrolimus TWBC, tacrolimus toxicity with nephrotoxicity, hyperglycemia and tremor were explained by a higher unbound fraction of tacrolimus.

After the tacrolimus dosage was adjusted on the basis of the predicted Hct-corrected TWBC, renal function began to recover and tremors decreased. Normal renal function was restored by day 30; insulin was progressively decreased and discontinued by day 41.

## Discussion

Oral absorption of tacrolimus is incomplete and influenced by food intake (Astellas Pharma US, 2012). Once absorbed, tacrolimus is primarily metabolized by CYP3A4/5 and distributed mainly into erythrocytes (Astellas Pharma US, 2012).

Tacrolimus is 85–98% bound to erythrocytes (Beysens et al., 1991; Trull, 1998; Steven, 2001). In plasma, tacrolimus is 99% bound, mainly to albumin and α-1-glycoprotein acid. This results in a free/unbound fraction in blood of less than 1% (Zahir et al., 2004). In addition to the type of transplanted organ, time since transplantation, age and sex, concomitant food consumption, changes in CYP3A4/5 activity, due to genetic polymorphisms or drug-drug interactions, as well as Hct levels have been shown to influence the PK of tacrolimus (Undre and Schäfer, 1998; Zhao et al., 2009; Staatz et al., 2010; Astellas Pharma US, 2012).

Tacrolimus is substrate of the P-gp, a multidrug efflux carrier expressed in many tissues, including the kidney, liver and intestine (Sikma et al., 2015). Intestinal P-gp has been shown to influence tacrolimus absorption (Masuda et al., 2005) while renal P-gp is not affecting tacrolimus blood concentrations but is rather linked to intracellular concentrations (Yan et al., 2016).

Tacrolimus metabolites are excreted 95% in the bile and only 2% in the urine (Möller et al., 1999). Although biliary obstruction or biloma could hypothetically impair biliary excretion of tacrolimus, reports in the literature are scarce and biliary flow has shown little effect on the PK of oral tacrolimus (Böttiger et al., 2002).

Tacrolimus TDM has been shown to be effective in reducing toxicity as well as preventing graft rejection (Staatz et al., 2001). Therapeutic tacrolimus target concentrations are based on empirical observations in adult transplant recipients. Because the concentration of the unbound fraction of tacrolimus represents the pharmacologically active drug and the fraction in equilibrium with the tissue concentration, unbound tacrolimus plasma concentrations would be the adequate surrogate for predicting clinical outcomes and dose adjustment. Measurement of total tacrolimus in plasma would be another possible alternative, as studies have shown a linear relationship between total and unbound tacrolimus plasma concentrations (Sikma et al., 2020). However, TWBC measurement is the gold-standard for tacrolimus TDM. The reasons for this are mainly technical: 1) The higher drug concentrations in whole blood than in plasma allows for easier quantification; 2) whole blood/plasma distribution ratio is affected by temperature; 3) plasma tacrolimus concentrations are vulnerable to hemolysis (Størset et al., 2014). In addition, to date, there are no validated methods or target concentrations for unbound or total tacrolimus in plasma (Machida et al., 1991; Sikma et al., 2020).

Numerous studies have previously identified Hct as a key factor in the interpretation of TWBC (Jusko et al., 1995; Størset et al., 2014; Sikma et al., 2020). As tacrolimus is a drug with a low hepatic extraction coefficient, when Hct increases, whole blood concentrations are expected to increase, with unbound concentrations remaining unchanged. With high Hct, downward adjustment of the dosage may result in decreased unbound concentrations and graft rejection (Zahir et al., 2004; Zahir et al., 2004). Conversely, low Hct levels may result in decreased whole blood concentrations without affecting the unbound plasma concentration (Undre and Schäfer, 1998; Zheng et al., 2012; Hebert et al., 2013; Størset et al., 2014; Sikma et al., 2020). The risk in this low Hct situation, is misinterpretation of subtherapeutic TWBC leading to an unnecessary or even dangerous increase in dose and toxicity (Sikma et al., 2020).

A few studies have developed PK models, equations and/or nomographs to integrate Hct in the interpretation of TWBC measurements (Størset et al., 2014; Sikma et al., 2020).

Storset et al. (2014) developed a population PK model and an equation to ‘normalize’ the TWBC to 45% Hct. In a paediatric study including 36 children (255 samples), Schijvens et al. (2019) developed an equation that integrates TWBC and Hct values, and transformed the usual whole blood target concentrations into *predicted target concentrations according to Hct*. They also developed a nomograph that defines the relationship between TWBC and plasma tacrolimus concentrations for different Hct and predicts the plasma tacrolimus concentration for different Hct.

Nephrotoxicity is a very common adverse effect of tacrolimus (Randhawa et al., 1997). It has been shown to correlate with the administered dose of tacrolimus, tacrolimus plasma concentrations above the TWBC and local renal exposure to tacrolimus (Bentata, 2020; Sikma et al., 2020). Tacrolimus overexposure also increases the risk of neurotoxicity, post-transplant diabetes, gastrointestinal complaints and hypertension (Zahir et al., 2004; Yuan et al., 2009; Stienstra et al., 2016).

The deterioration of renal function frequently observed in patients after transplantation is most often multifactorial, favored by sepsis, hypovolemia, inflammatory phenomena and the administration of nephrotoxic agents such as tacrolimus (Sharma et al., 2009; Leroy et al., 2010); this is most likely the case in our patient who was hypovolemic and had received a contrast agent. Furthermore, with regard to tacrolimus, in addition to the high doses she had received, *ABCB1* genetics of the donor may have affected the risk of nephrotoxicity. The *ABCB1* CT c.3435 predicts reduced P-gp functionality at the graft level and thus could theoretically have participated in the intraparenchymal accumulation of tacrolimus (Hauser et al., 2005). Although still controversial in the literature, this variant has been associated with decreased renal function at 1, 3 and 6 months and 1 year after transplantation (*p* < 0.01) when the graft carries the CT or TT genotypes compared with CC (Tavira et al., 2015; Sallustio et al., 2021).

Determination of tacrolimus plasma concentrations in our patient could have prevented dose increase and involvement in acute nephrotoxicity, as well as the development of hypertension, hyperglycemia and neurotoxicity.

## Conclusion

Our case report illustrates how low Hct can lead to misinterpretation of subtherapeutic tacrolimus concentrations, subsequently leading to a dangerous dose increase and risk of toxicity.

Due to the absence of analytical methods to measure unbound tacrolimus, as well as validated methods to determine the Hct-corrected whole blood concentration, the gold standard for TDM remains the measurement of TWBC. In practice, when prescribing tacrolimus, the risk of toxicity in case of low Hct should be kept in mind, even when TWBC are within target concentrations. In situations of low Hct, we recommend aiming for the lower therapeutic level.

## Data Availability

The original contributions presented in the study are included in the article/supplementary material, further inquiries can be directed to the corresponding author.

## Abbreviations

CNI, calcineurin inhibitors; CYP, Cytochrome P450; Ct, trough concentration; ECLIA, Electro-chemiluminescence Immunoassay; Hct, hematocrit; IV, intravenous; KDIGO, kidney disease improving global outcome; LC/MS, Liquid chromatography-mass spectrometry; P-gp, P-glycoprotein; PK, pharmacokinetic; TDM, Therapeutic drug monitoring; TWBC, Tacrolimus whole blood concentration.
